# Entrepreneurial education and its role in fostering sustainable communities

**DOI:** 10.1038/s41598-024-57470-8

**Published:** 2024-03-30

**Authors:** M. Suguna, Aswathy Sreenivasan, Logesh Ravi, Malathi Devarajan, M. Suresh, Abdulaziz S. Almazyad, Guojiang Xiong, Irfan Ali, Ali Wagdy Mohamed

**Affiliations:** 1 School of Computer Science and Engineering, Vellore Institute of Technology, Chennai, 600127 India; 2 Amrita School of Business, Amrita Vishwa Vidyapeetham, Coimbatore, 641112 India; 3 Centre for Advanced Data Science, Vellore Institute of Technology, Chennai, 600127 India; 4 School of Electronics Engineering, Vellore Institute of Technology, Chennai, 600127 India; 5 Department of Computer Engineering, College of Computer and Information Sciences, King Saud University, P.O. Box 51178, 11543 Riyadh, Saudi Arabia; 6 Guizhou Key Laboratory of Intelligent Technology in Power System, College of Electrical Engineering, Guizhou University, Guiyang, 550025 China; 7 Department of Statistics and Operations Research, Aligarh Muslim University, Aligarh, 202002 India; 8 Operations Research Department, Faculty of Graduate Studies for Statistical Research, Cairo University, Giza, 12613 Egypt

**Keywords:** Entrepreneurship education, Sustainable communities, Sustainable development goals, Diverse community development, Total interpretive structural modeling, Sustainability, Socioeconomic scenarios

## Abstract

Establishing sustainable communities requires bridging the gap between academic knowledge and societal requirements; this is where entrepreneurial education comes in. The first phase involved a comprehensive review of the literature and extensive consultation with experts to identify and shortlist the components of entrepreneurship education that support sustainable communities. The second phase involved Total Interpretative Structural Modelling to explore or ascertain how the elements interacted between sustainable communities and entrepreneurial education. The factors are ranked and categorized using the Matrice d'impacts croises multiplication appliquee an un classement (MICMAC) approach. The MICMAC analysis classifies partnerships and incubators as critical drivers, identifying Student Entrepreneurship Clubs and Sustainability Research Centers as dependent elements. The study emphasizes alumni networks and curriculum designs as key motivators. The results highlight the critical role that well-designed entrepreneurial education plays in developing socially conscious entrepreneurs, strengthening communities, and generating long-term job prospects. The study provides a valuable road map for stakeholders dedicated to long-term community development agendas by informing the creation of strategic initiatives, curriculum updates, and policies incorporating entrepreneurial education.

## Introduction

Sustainability represents a fresh way of reframing the interaction between people and the natural world, making it more than merely a research topic. It emphasizes how inadequate environmental protection is on its own. Instead, pursuing sustainability necessitates looking beyond self-interest and addressing social and economic aspects^1,2^. Thinking ahead means ensuring the next generation has at least as many opportunities as the current one^3^. The next generation's new models for balancing ecosystems—which combine socioeconomic development and environmental protection—may be sparked by sustainable education and trust^4^. Sustainable development is critical to our shared future, and entrepreneurship is acknowledged as a powerful driver of this sustainable economic growth^5,6^. In order to thrive without sacrificing future demands, modern businesses are aggressively implementing sustainability concepts^7^. Entrepreneurial skills are needed to address sustainability's social, economic, and environmental aspects and overcome challenges in today's rapidly evolving global world. The significance of entrepreneurial education in preparing people for sustainability-focused projects is shown by the emphasis on the entrepreneurial spirit in the shift to sustainable communities^8^. Smart cities (SC) should implement specific measures to prevent isolation in academic institutions, thereby fostering the formation of community clusters. Furthermore, since encouraging immigrant entrepreneurship can boost the local economy, SC should lessen the difficulties associated with starting a firm and providing mentorship and training to entrepreneurs involved in administration and regulation^9^.

Recent research underscores the significance of integrating sustainability into entrepreneurial education. Kotla and Bosman^10^ argue for a multifaceted strategy to bridge the gap between integrating sustainability and entrepreneurship in higher education. The difficulties arise from the necessity of fusing the long-term, systemic perspective required by sustainability with the dynamic, frequently unpredictable character of entrepreneurship. In light of the numerous and intricate difficulties we face today, Klapper and Fayolle^11^ suggest redefining entrepreneurial education to effectively address sustainability, social justice and hope. In order to assist with the purpose of sustainability, Fanea-Ivanovici and Baber^12^ look into how colleges may help Indian students who aspire to be future entrepreneurs by promoting sustainability and sustainable development goals.

As a multifaceted approach, entrepreneurial education fosters creativity, adaptability, and a profound understanding of socioeconomic dynamics^13^. It explores the profound effects of an entrepreneurial mindset on social structures, environmental preservation, and long-term economic sustainability in society, going beyond traditional business acumen. This study's main objective is to investigate the variables that influence how entrepreneurship education contributes to the development of sustainable communities.

Although there is a growing corpus of research examining the distinct effects of sustainable community development and entrepreneurial education^6,14,15^, a thorough grasp of the complex interactions between these two fields is noticeably lacking. This research gap highlights the need for a study that uses a systematic modeling method to reveal the complex linkages between sustainable community development and entrepreneurial education and explore the individual contributions of these two phenomena. By providing a novel on the practical ways in which entrepreneurial education may support sustainable community development, this study aims to close this gap. Based on the latest developments in sustainability research and entrepreneurship education, our method uses Total Interpretive Structural Modeling (TISM) to methodically examine the intricate connections between these fields. Our research attempts to provide detailed knowledge of how entrepreneurial education might encourage sustainable community development in various socioeconomic circumstances by identifying essential components and their interdependencies. The novelty of our study resides in its theoretical framework and methodological approach, which combine ideas from the most recent literature with empirical analysis to provide practitioners, policymakers, and educators with helpful information. We contribute to the theoretical debate on sustainable entrepreneurial education by synthesizing and expanding on existing research and providing helpful advice for creating successful educational initiatives and policy interventions.

TISM, particularly influential within the context of startups, is employed in this study to answer the following research questions: “What are the factors influencing the role of entrepreneurship education in fostering sustainable communities? How do they influence one another and entrepreneurship education in fostering sustainable communities? Which factors drive others, and which factors depend on others? Can the priority of each of these factors be measured?”

Our study takes a two-pronged approach, starting with a thorough qualitative analysis to pinpoint the variables influencing the contribution of entrepreneurship education to sustainable community development. To provide a balanced view, we also consulted a review of the literature and expert comments. After that, we move into a quantitative phase where we use TISM to methodically investigate the interactions between these components, revealing their hierarchical structure and effects on the entrepreneurial education ecosystem. This mixed-methods approach guarantees a comprehensive analysis by combining quantitative clarity with qualitative depth to shed light on the intricate dynamics involved in utilizing entrepreneurship education for sustainable community development.

Table 1 presents the identified factors influencing the role of entrepreneurship education in fostering sustainable communities:

**Table 1:** Factors influencing the role of entrepreneurship education.

Factor number	Factor	References
F1	Curriculum design and relevance	^16^
F2	Sustainability research centers	^17^
F3	Alumni network	^18^
F4	Community outreach programs	^19^
F5	Entrepreneurship as job creators	^20^
F6	Urban entrepreneurship initiatives	^21^
F7	Globalization and exchange programs	^22^
F8	Student entrepreneurship club	^23^
F9	Partnership with local businesses	^24^
F10	Incubators and accelerators	^25^

This paper is structured as follows: The research approach is discussed in the subsequent section, presenting findings and discussions. Subsequently, the paper depicts managerial/practical, theoretical, and societal contributions while finally including the conclusion, limitations, and future study areas.

## Research methodology

In order to assess the influence of the identified enablers, this study uses a closed-ended questionnaire with pairwise comparisons^26^. Semi-structured interviews provide detailed insights because they are exploratory in character^27^. Data analysis techniques include TISM and MICMAC analyses. The study use snowball sampling to identify participants aware of the importance of entrepreneurship education in sustainable communities. Prioritizing convenience over ethical considerations led to conducting one-hour company interviews over a month. Twenty-seven Indian entrepreneurs from various industries and areas participated in the study. Participants had various experiences and viewpoints because they were involved in different business endeavors. Convenience played a role in participant selection, but ethical considerations came first. The study proactively ensured adherence to the highest ethical standards by implementing necessary measures. It sought informed consent to prioritize participants' autonomy by outlining the study's goals and ensuring voluntary participation. Strict protocols protected confidentiality and privacy; personally identifying information was securely managed, available only to the research team, and never revealed in published data or conclusions. These ethical protections highlight the dedication to participant welfare and scientific integrity throughout the study. The closed-ended survey consists of broad and specific questions that are scored on a five-point Likert scale to determine how different elements affect the development of sustainable communities. A TISM and MICMAC are employed to identify the prominent, influential relations amongst entrepreneurship education's contribution to sustainable communities.

### Data analysis method

**Figure 1:**
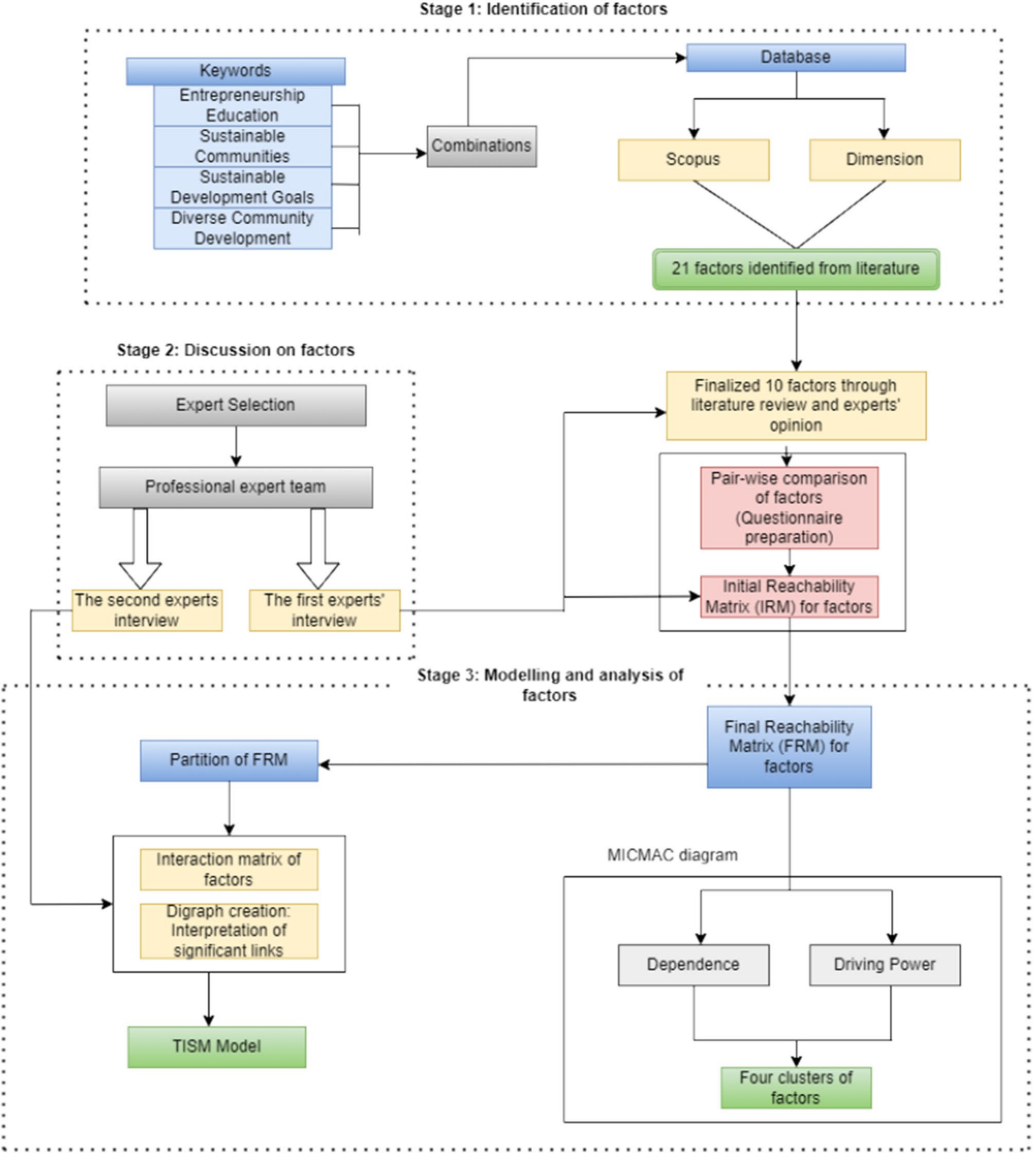
Flow of TISM approach for entrepreneurship education and its role in fostering sustainable communities.

Figure 1 shows the steps in the research approach sequence. The conventional ISM approach, which creates a contextual relationship-based performance framework, is expanded upon by TISM^28^. The detected components and their associated order structure are displayed in the structural model created using the TISM methodology by their reciprocal influencing relationships^29^. The TISM technique facilitates the modeling of interrelationships between variables in a digraph form. An arrow represents the flow and hierarchical order of the relationships between the elements. The connecting arrow denotes the contextual connections between any two elements, and the levels at which the significant aspects are ultimately organized in the diagram define the influencing factors. TISM builds the model by considering just the most useful transitive relationships and leverages expert input to confirm the trustworthy source of transitivity, if any. In line with the approaches taken by other researchers, this study models entrepreneurship education variables and their function in creating sustainable communities using TISM^30^ (Jayalaksmi & Pramod, 2015). TISM modeling commences with the critical task of identifying and defining the components for analysis.

The study determines the critical components of entrepreneurship education contribution to sustainable communities through a survey of the literature and expert discussions. During our literature evaluation, we carefully examined every peer-reviewed article released in the past. This comprehensive research aimed to gather various viewpoints regarding the effects, modes of operation, and results of entrepreneurship education about sustainable community development. We held expert discussions after the literature review to deepen our comprehension of the crucial elements found. We investigated their perspectives on successful teaching strategies, obstacles encountered when including sustainability in entrepreneurship education, and possible long-term effects on communities through semi-structured interviews. Through these discussions, we could confirm our conclusions from the literature review and pinpoint any new themes or neglected regions. By integrating findings from expert talks and the literature review, we developed a comprehensive and evidence-based framework that outlines the essential elements of entrepreneurship education that support sustainable communities. Table 1 displays ten components and pertinent references chosen from a list of twenty-one. After identifying factors, the next step is to ascertain the contextual connections among these elements. Subject matter experts offer perspectives that shed light on these linkages. These connections within the framework suggest that “factor A influences or improves factor B.” Based on experts' judgments, a “pairwise interaction matrix” is created to show the interactions between the elements.

TISM goes above and beyond Interpretive Structural Modelling (ISM) by elucidating these linkages' mechanisms. A high influence is denoted by a 1 in the Initial Reachability Matrix (IRM) (Table 2), whereas a low influence is indicated by a 0. The Final Reachability Matrix (FRM) was created by appending the “transitivity rule” to the IRM (Table 3). Following transitivity testing, the transitive elements—represented by the number “0” in the IRM—are replaced with “1*” in the FRM. Organizing components level by level is the next stage. With other influencing factors, variables comprise the “antecedent set,” each factor's “reachability set” consists of further elements it might affect. For every aspect, the “intersection set” is found. The element-sharing entities with the “intersection set” and the “reachability set” are advanced to the top level in each iteration. The study repeats this process until all element levels are determined. The “interaction matrix design” is shown in Table 4.

**Table 2:** IRM for factors influencing entrepreneurial education.

	F1	F2	F3	F4	F5	F6	F7	F8	F9	F10
F1	1	1	0	1	1	1	0	0	1	0
F2	0	1	0	0	0	0	0	1	0	0
F3	1	1	1	0	1	1	1	1	1	1
F4	1	0	0	1	1	1	0	0	1	0
F5	0	0	0	0	1	0	0	0	0	0
F6	0	1	0	0	0	1	0	1	1	1
F7	0	1	1	1	0	0	1	0	0	0
F8	0	1	0	0	1	0	0	1	0	0
F9	0	1	0	0	1	1	0	0	1	1
F10	0	0	0	0	1	1	0	1	1	1

*Represents transitive links.

^#^Represents significant transitive links.

**Table 3:** FRM for factors influencing entrepreneurial education.

	F1	F2	F3	F4	F5	F6	F7	F8	F9	F10	Driving power
F1	1	1	0	1	1	1	0	1*	1	1*	8
F2	0	1	0	0	1*	0	0	1	0	0	3
F3	1	1	1	1*	1	1	1	1	1	1	10
F4	1	1*	0	1	1	1	0	1*	1	1*	8
F5	0	0	0	0	1	0	0	0	0	0	1
F6	0	1	0	0	1*	1	0	1	1	1	6
F7	1*	1	1	1	1*	1*	1	1*	1*	1*	10
F8	0	1	0	0	1	0	0	1	0	0	3
F9	0	1	0	0	1	1	0	1*	1	1	6
F10	0	1*	0	0	1	1	0	1	1	1	6
Dependence	4	9	2	4	10	7	2	9	7	7	

*Represents transitive links.

**Table 4:** Interaction matrix.

	F1	F2	F3	F4	F5	F6	F7	F8	F9	F10
F1	1	1	0	1	1	1	0	0	1	0
F2	0	1	0	0	1^#^	0	0	1	0	0
F3	1	1	1	1^#^	1	1	1	1	1	1
F4	1	0	0	1	1	1	0	0	1	1^#^
F5	0	0	0	0	1	0	0	0	0	0
F6	0	1	0	0	1^#^	1	0	1	1	1
F7	1^#^	1	1	1	0	0	1	1^#^	0	1^#^
F8	0	1	0	0	1	0	0	1	0	0
F9	0	1	0	0	1	1	0	1^#^	1	1
F10	0	0	0	0	1	1	0	1	1	1

^#^Represents significant links.

**Figure 2:**
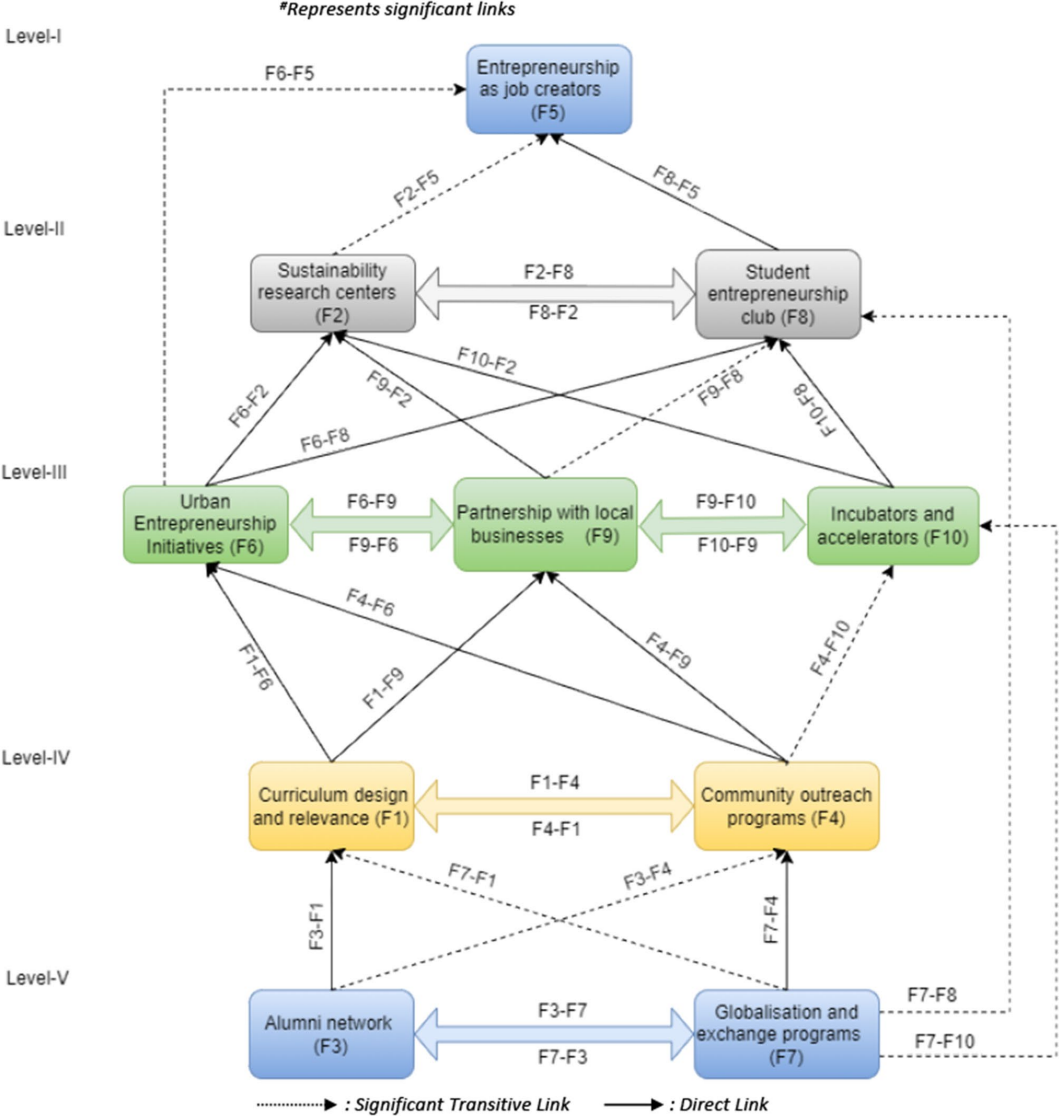
TISM model for factors influencing entrepreneurial education.

A directed graph (digraph) is produced by visually organizing the elements based on their levels and connecting them through the linkages found in the FRM. The digraph includes all “transitive links” and provides insightful explanations. Every relationship in TISM is defined and explained logically. Developing interpretive assertions about the digraph's links is part of this process. The study then utilizes the data to construct the TISM model (Fig. 2) by replacing the factors with the digraph nodes.

### Ethical considerations

The Amrita Vishwa Vidyapeetham (AVV) institutional review board approved the study, and we obtained a formal letter of permission from Amrita Vishwa Vidyapeetham, the school of business, with registration number ERB-ASB-2023-020. There is no potential risk that may cause any harm to respondents. The procedures used in this study adhere to the tenets of the Declaration of Helsinki.

### Ethical approval and consent to participate

The study received ethical approval from the AVV Ethical Review Committee and written informed consent from each participant. The study ensured that all methods complied with relevant guidelines and regulations.

## Results

### Interpretation of TISM digraph

Figure 2 visually depicts the TISM analysis of factors influencing entrepreneurial education and its role in promoting sustainable communities, while Table 5 provides an interpretation of the findings.

**Table 5:** Synthesis of the interpretation of the TISM graph.

Levels	Influence factor	Influencing factor	Key impact mechanism
V	F3	F1	Through a constant feedback loop, alumni's critical analysis improves the relevancy of the curriculum and allows for continued changes. Alumni's practical experiences guide course design^31^
		F2	In order to support the advancement of the upcoming generation of sustainability experts, alumni mentor researchers pursuing sustainable careers^32^
		F4	Alumni encourage community involvement and social responsibility Participating in voluntary work enhances learning opportunities Alumni use training sessions to share their expertise^33^
		F5	Serve as role models and mentors for future entrepreneurs Create jobs and boost the economy through alumni enterprises
		F6	Alumni entrepreneurs offer insightful opinions on urban projects
		F7	The alumni network promotes worldwide cooperation and globalization It also makes cross-cultural dialogue and comprehension easier
		F8	Networking possibilities through alumni contacts Mentorship programs for student entrepreneurs^34^
		F9	Alumni networks strengthen commercial ties with the community Give them access to industry insights and professional networks
		F10	Exposure and networking possibilities for companies Guidance and advice for companies residing in incubators
	F7	F1	Adds a variety of international viewpoints to the program Stresses intercultural communication in the classroom
		F2	Enhances collaboration and aligns research with international sustainability objectives^35^
		F3	A varied alumni base raises the value of the worldwide network Programs for cultural interaction help graduates understand one another better
		F4	Initiatives for community engagement are enhanced by cultural diversity Encourages intercultural dialogue and global citizenship
		F8	Adds diversity to student-run business organizations Promotes initiatives for cultural entrepreneurship and cross-cultural interaction
		F10	Makes it easier for startup ecosystems to go global Promotes international networking and mentoring opportunities
IV	F1	F2	Research objectives and operations of sustainability research centers are impacted by curriculum design prioritizing sustainability^36^
		F4	Curriculum design courses incorporating service learning facilitate community engagement programs^37^ Helps students acquire competencies related to community outreach Curriculum inclusion for inclusive community outreach that includes diversity and cultural competency
		F5	Emphasizing entrepreneurial qualities in curricula helps students prepare to start businesses and create jobs Including sustainable business strategies in the socially conscious entrepreneurship program
		F6	The objectives of Urban Entrepreneurship Initiatives are aligned with curriculum design that focuses on urban challenges Urban entrepreneurship is supported when community involvement is given priority in the curriculum
		F9	CSR and community involvement are included in the curriculum to produce socially responsible workers^38^ Projects carried out in conjunction with nearby companies improve community effect A curriculum designed to accommodate the demands of local businesses benefits local businesses and students
	F4	F1	Curriculum is influenced by outreach program insights, which include social responsibility and ethical issues
		F5	Workshops on entrepreneurial skills offered as part of community outreach initiatives help to create jobs Programs like business incubators and accelerators help new businesses and the creation of jobs Providing microenterprise support services and microfinance initiatives help create jobs locally
		F6	Community involvement activities shape urban entrepreneurship initiatives Encourages economic expansion and a healthy atmosphere and solves issues that urban enterprises encounter Promotion of legislation encouraging urban business Helps entrepreneurs overcome regulatory challenges
		F9	Impact on forming trust, identifying requirements, and comprehending the local business environment about relationships with local businesses Promoting networking and communication opportunities Cooperation with a focus on shared objectives and community needs Increased company visibility and assistance for a supportive environment
		F10	Incubators and accelerators can reach a wider audience through community outreach Promotes strategic partnerships by deepening contacts with regional organizations Aligns initiatives with local objectives and assists business owners with difficulties Creates an environment that is conducive to innovation hubs
III	F6	F2	Sustainability Research Centers can gain practical, idea-based insights from urban entrepreneurship projects
		F5	Urban entrepreneurship initiatives stimulate local businesses' growth and employment generation Emphasize creating highly skilled jobs in the creative, innovative, and technological sectors Participation from the community in urban entrepreneurial projects creates jobs locally
		F8	Student entrepreneurship clubs can access networks and real-world learning experiences through participation in urban entrepreneurship projects Through partnerships with businesses, accelerators, and incubators, students are exposed to the intricacies of urban entrepreneurship Forums for collaboration between student entrepreneurs and established urban firms are established
		F9	Urban entrepreneurship initiatives give nearby businesses a forum to interact and promote budding entrepreneurs Using ingenuity and creativity to draw new companies to the neighborhood economy The local business ecosystem's economic vibrancy is improved Cooperation between established local firms and expanding urban enterprises is beneficial
		F10	By developing ecosystems, encouraging networking, identifying startup requirements, and establishing a steady flow of creative businesses, urban entrepreneurship programs improve incubators and accelerators and benefit the larger urban entrepreneurial scene (Madaleno et al., 2022)
	F9	F2	Through relationships with local businesses, sustainability research centers obtain useful information and business insights that enhance their comprehension of environmental impacts and enable them to apply empirical data from area business operations in real-world scenarios
		F5	By establishing partnerships with neighboring businesses, increasing success rates, creating jobs, and broadening the employment market, entrepreneurs bolster their endeavors. They also develop specialty markets in conjunction with helpful local businesses
		F6	Through collaborations that benefit established and growing businesses, urban entrepreneurship initiatives promote local business contact, increasing economic growth, resource access, and the broader metropolitan economic fabric
		F8	In order to create a win–win collaboration where students assist local businesses and corporations in mentoring budding entrepreneurs, local company relationships provide student entrepreneurial clubs with networking opportunities and mentorship Strengthens and maintains the community of local entrepreneurs
		F10	Local company relationships supplement incubators and accelerators with market validation, corporate sponsorship, investment, networking, industry expertise, and economic effect through various channels
	F10	F5	Incubators and accelerators facilitate the creation of jobs for growing firms by offering resources, networking opportunities, and mentorship^39^ Through hands-on learning, networking events, mentorship, and workshops, entrepreneurs hone their talents
		F6	By bolstering the ecosystem, offering physical spaces, coaching, and support for urban goals, incubators, and accelerators impact urban entrepreneurship^40^ They act as avenues for applying creative thinking and entrepreneurial skills to specific urban problems, providing direct lines of communication for urban entrepreneurship efforts
		F8	Incubators and accelerators help student entrepreneurial groups by providing experience, guidance, and practical knowledge^41^ These partnerships foster a vibrant learning environment that welcomes practical initiatives that give students instruction in strategy, marketing, and product development
		F9	Incubators and accelerators connect startups with local ones, creating a collaborative environment Innovation centers encourage collaboration, increase startup awareness, and indicate that a project is ready for joint venture success
II	F2	F5	Research centers focused on the impact of sustainability on entrepreneurship by encouraging environmentally conscious behaviors and technologies that support robust business growth and job creation^42^ Using information from these hubs, entrepreneurs construct socially and ecologically responsible companies supporting green jobs and sustainable practices
		F8	Student entrepreneurial clubs receive guidance on sustainable entrepreneurship, eco-friendly practices, and tactics from sustainability research organizations, which helps to shape their viewpoints and methods
	F8	F2	Similar to student clubs, entrepreneurship groups seek to spot and seize emerging business trends. By bringing attention to fresh subjects or problems in sustainable entrepreneurship, they want to impact sustainability centers' research objectives
		F5	Student-run entrepreneurship clubs promote creativity, opportunity recognition, and prudent risk-taking by developing an entrepreneurial mentality These clubs foster an environment favorable to starting new companies, producing jobs, and highlighting social entrepreneurship for societal benefit through events, workshops, and networking
I	F5		Entrepreneurship as job creators (F5) is related to the objective of this study

### MICMAC analysis

Compared to previous multi-attribute techniques, TISM has several advantages, but it still cannot analyze the strength and relationship between the components. MICMAC addresses this TISM problem by categorizing the relationships between the components to make the concept of driving and dependency power more understandable. It also distinguishes between strong and weak elements since their interactions are not consistently balanced and can alter in response to environmental demands^43^. The MICMAC framework identifies four main zones as elements associated with entrepreneurial education: autonomous factors, dependent factors, linkage factors, and driving (independent) factors. The following are each zone’s characteristics:

1. Autonomous factors (Zone 1): These are known as autonomous enablers with weak reliance and
2. Driving power^44^. Notably, this study’s components do not fall under this autonomous zone.
3. Dependence factors (Zone 2): We classify these variables as dependence factors because other variables strongly depend on them but have a lower driving force^45^.
4. Linkage factors (Zone 3): Linkage factors are those that exhibit both firm reliance and strong driving power and driving or independent factors: These are what are known as driving or independent factors since they have a significant driving force in curriculum design and relevance, and community outreach initiatives are among the motivating elements found in this study.
5. Driving factors (Zone 4): These variables are referred to as driving factors since they strongly drive the other variables but have a lower dependence^45^. Table 6 presents the ranking of the elements impacting entrepreneurial education based on the MICMAC analysis.

**Table 6:** MICMAC ranks for factors influencing entrepreneurial education.

Factor	Driving power	Dependence	Driving power/dependence	MICMAC rank
F1	8	4	2.000	2
F2	3	9	0.333	4
F3	10	2	5.000	1
F4	8	4	2.000	2
F5	1	10	0.100	5
F6	6	7	0.857	3
F7	10	2	5.000	1
F8	3	9	0.333	4
F9	6	7	0.857	3
F10	6	7	0.857	3

**Figure 3:**
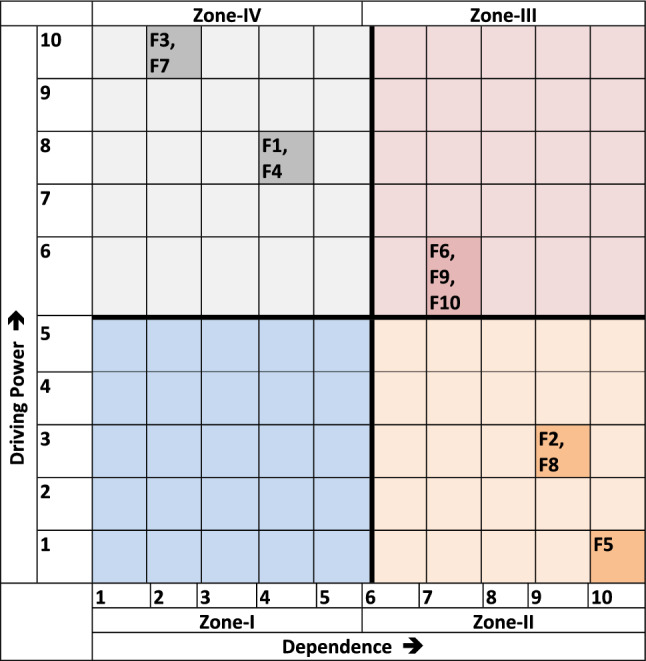
MICMAC graph.

To illustrate the MICMAC analysis, Fig. 3 presents the corresponding graph. Based on its driving force and dependence, Table 5 ranks the variables impacting entrepreneurial education and its function in developing sustainable communities. The rankings place globalization and exchange programs (F7) and alumni networks (F3) at the top. The MICMAC analysis ranks entrepreneurship as job producers (F5) at the fifth position. It indicates a greater reliance on external factors.

## Discussion and implications

The complex web of interconnected components that make up entrepreneurial education emphasizes the need for a comprehensive approach to promote long-term community development. This conversation explores the consequences of our research and compares it with existing literature to highlight how vital entrepreneurial education is in promoting sustainable behaviors in various contexts.

Our research points to sustainable education as a crucial component, consistent with the body of literature highlighting the transformational potential of education in fostering sustainable practices and beliefs^46^. In line with UNESCO's emphasis on Education for Sustainable Development, entrepreneurial education incorporating sustainability into the curriculum generates socially and environmentally conscious entrepreneurs and sparks creative solutions to urgent global issues^47^.

Our research demonstrates how entrepreneurial education can foster inclusive and resilient economic growth, and the study highlights workable solutions for achieving the Sustainable Development Goals through entrepreneurship. These solutions include encouraging urban entrepreneurship and supporting incubators and accelerators, which are approaches backed by research on the vital role of entrepreneurship in sustainability. Our study proposes a balanced strategy integrating social equality, economic viability, and environmental stewardship into the entrepreneurial education ecosystem while adopting a pragmatic sustainability perspective. This concept is consistent with the triple bottom line approach—which takes sustainability to include social, environmental, and economic aspects—discussed in the literature^48^. Focusing on a practical approach emphasizes how important it is for entrepreneurial education programs to equip students with the skills they need to traverse and balance various dimensions successfully.

Our study's findings, which highlight the interdisciplinarity in successful entrepreneurial education programs, emphasize how critical it is to transcend conventional academic boundaries to handle challenging sustainability issues. Literature emphasizing the importance of interdisciplinary approaches in generating innovation and problem-solving abilities required for sustainable development supports this finding^49^.

Our research actively highlights the importance of stakeholder engagement in strengthening the ecosystem of entrepreneurial education for sustainability. It is consistent with research showing that partnerships, local expertise, and a better understanding of community needs are all made possible through stakeholder participation, strengthening educational efforts' resilience and sustainability.

This study, which enriches theories by analyzing the effect of entrepreneurship education on sustainable community development, uses TISM as a methodological framework. The results highlight how entrepreneurial education can support socially conscious behavior and support comprehensive strategies for long-term community sustainability.

By emphasizing sustainability, entrepreneurial education helps underprivileged populations become more powerful, which lowers inequality and promotes inclusive economic growth. This socially responsible strategy fosters the development of a new generation of company leaders, encouraging moral behavior and long-term job creation. It improves civic engagement, community resilience, and environmental stewardship. Promoting sustainable habits in society and stimulating innovation are two benefits of entrepreneurial education that may extend to public health. In conclusion, including sustainability in education has long-term advantages that range from enhanced quality of life to social cohesion and economic development.

With its practical implications, this study substantially improves community sustainability via entrepreneurial education. Specific implications and additions to the sustainability of communities are:

- Program designers and instructors should take a comprehensive approach to creating and executing entrepreneurial education initiatives. Understanding how these elements are related to one another is essential for a thorough and successful educational plan.
- Educational institutions and support networks must prioritize adaptability and ongoing observation. Ensuring the robustness of dependent factors through responsiveness to environmental changes sustains the efficacy of entrepreneurial education programs.
- These linkage elements (i.e., initiatives promoting urban entrepreneurship, alliances with nearby companies, incubators, and accelerators) should be actively supported and funded by policymakers and local government units. Acknowledging their critical role in establishing strong connections inside the system creates an atmosphere favorable for long-term entrepreneurial endeavors.
- Educators and policymakers should prioritize the driving factors when creating and executing programs for entrepreneurial education.
- Highlighting these elements strengthens the overall effectiveness and success of community-focused entrepreneurship projects

General contributions to the sustainability of the community are:

- Policymakers, educators, and support groups can use the study's findings to build entrepreneurial education programs tailored to sustainable community development.
- The recommendations include redesigning courses, judiciously assigning resources, and offering educators specialized training. These actions enhance the effectiveness and long-term success of entrepreneurship education programs.
- Local government agencies and development organizations should collaborate by aligning their initiatives with educational objectives. For businesses and entrepreneurs, ongoing evaluations offer insightful information that promotes long-term success.
- Including sustainability as a critical education component empowers marginalized groups, lowers inequality, encourages inclusive economic growth, and develops socially conscious behavior.
- The socially responsible approach fosters a new generation of socially conscious leaders enabled through entrepreneurial education. It also increases civic involvement, community resilience, and environmental stewardship.

## Conclusion

Investigating the complex interactions between entrepreneurship education and its influence on developing sustainable communities is crucial, as demonstrated using TISM and MICMAC analysis. The understanding of entrepreneurship education as a complex ecosystem with interdependent parts that work together to achieve sustainability is the fundamental tenet of our research. The meticulous mapping of these elements has shed light on the ecosystem's dynamism and complexity, exposing a web of interrelationships that support the idea that entrepreneurship education can support the establishment of sustainable communities.

Through TISM and MICMAC analysis, this study explores the function of entrepreneurship education in promoting sustainable communities. Using this method, we could map the roles and relationships of 10 critical components of the entrepreneurship education ecosystem. It improved the comprehension of how these components work together to affect sustainability. According to our analysis, every element in the entrepreneurship education ecosystem actively works to create sustainable communities; not a single element operates independently. It demonstrates the intricate nature of the ecosystem, in which each element—including dependent elements like student entrepreneurial groups and sustainability research centers—plays a vital role. These dependent components highlight the interconnectedness of the ecosystem and the need for supportive interactions to meet sustainability goals. Their distinction lies in their low driving strength and high dependence on influence from more dominant forces. Our findings reveal a startling fact: the entrepreneurial education ecosystem is incredibly intertwined. Each component is essential to the general health and effectiveness of the ecosystem, meaning that this interconnection is not just structural but also functional. Identifying interdependent components, such as sustainability research centers and student entrepreneurial clubs, highlights the delicate balance of the ecosystem, where the vitality of its constituent parts influences the resilience and flexibility of the whole.

The study emphasizes the significance of driving forces and linking factors as crucial components that create connections and advance the ecosystem. Establishing connections with nearby businesses, initiatives promoting urban entrepreneurship, and the thoughtful planning of the curriculum are essential for connecting the many components of the ecosystem and focusing their combined efforts on achieving lasting results. The results above highlight the need for a systematic and comprehensive strategy to improve the ecosystem of entrepreneurship education, stressing the vital functions of stakeholder collaboration, curricular relevance, and community involvement.

Linkage variables show a substantial dependence on other factors and significantly influence them. Examples of these are relationships with local firms and urban entrepreneurship efforts. These constituents are crucial in connecting disparate elements of the entrepreneurship education framework, guaranteeing a unified and cooperative endeavor to cultivate sustainable communities.

Through integrating TISM to examine variables affecting sustainable community development, this study promotes ideas related to entrepreneurship education. Theoretical ramifications include developing comprehensive frameworks for sustainability and improving social entrepreneurship ideas. Practical applications guide governments, entrepreneurship organizations, educational institutions, and community leaders. Opportunities for inclusive employment, socially responsible company practices, and community empowerment are among the societal effects. However, the dynamic nature of entrepreneurial ecosystems and findings particular to a given setting are limits. The paper provides complementary strategies for future research and advises caution when extrapolating results. The study offers a comprehensive grasp of the variables in entrepreneurial education. However, it also calls for more investigation into contextual variations, longitudinal impacts, the effectiveness of interventions, and regional/cultural influences. Overall, it emphasizes how entrepreneurship education can be revolutionary when it aligns with environmental goals and helps create sustainable communities and resilient economies.

Although our study offers insightful information about the connection between sustainable community development and entrepreneurship education, it is important to recognize several limitations that could impact how our findings are interpreted and applied more broadly. Due to the specific environment of this study, its conclusions might only apply to some situations or demographics. Cultural variations, economic conditions, and educational systems contribute to distinct effects on the dynamics of entrepreneurship education and its impact on community sustainability across different contexts. New ideas, regulations, and methods are constantly emerging in sustainable community development and entrepreneurial education. Although our analysis offers a quick overview of the situation, it might not account for long-term patterns or upcoming advancements in the sector. Given these limitations, it is essential to interpret the results with caution and to remember that further research is needed to examine these correlations in greater detail using a variety of approaches, contexts, and sample sizes. Our intention in disclosing these limitations is to foster openness and stimulate thoughtful consideration of the extent and consequences of our research.

## Data Availability

The datasets used and/or analyzed during the current study are available from the corresponding author on reasonable request.
